# High sensitivity ^1^H-NMR spectroscopy of homeopathic remedies made in water

**DOI:** 10.1186/1472-6882-4-15

**Published:** 2004-11-01

**Authors:** David J Anick

**Affiliations:** 1 Harvard Medical School Mailman Building 123, McLean Hospital, 115 Mill St., Belmont, MA 02478, USA

## Abstract

**Background:**

The efficacy of homeopathy is controversial. Homeopathic remedies are made via iterated shaking and dilution, in ethanol or in water, from a starting substance. Remedies of potency 12 C or higher are ultra-dilute (UD), i.e. contain zero molecules of the starting material. Various hypotheses have been advanced to explain how a UD remedy might be different from unprepared solvent. One such hypothesis posits that a remedy contains stable clusters, i.e. localized regions where one or more hydrogen bonds remain fixed on a long time scale. High sensitivity proton nuclear magnetic resonance spectroscopy has not previously been used to look for evidence of differences between UD remedies and controls.

**Methods:**

Homeopathic remedies made in water were studied via high sensitivity proton nuclear magnetic resonance spectroscopy. A total of 57 remedy samples representing six starting materials and spanning a variety of potencies from 6 C to 10 M were tested along with 46 controls.

**Results:**

By presaturating on the water peak, signals could be reliably detected that represented H-containing species at concentrations as low as 5 μM. There were 35 positions where a discrete signal was seen in one or more of the 103 spectra, which should theoretically have been absent from the spectrum of pure water. Of these 35, fifteen were identified as machine-generated artifacts, eight were identified as trace levels of organic contaminants, and twelve were unexplained. Of the unexplained signals, six were seen in just one spectrum each. None of the artifacts or unexplained signals occurred more frequently in remedies than in controls, using a p < .05 cutoff. Some commercially prepared samples were found to contain traces of one or more of these small organic molecules: ethanol, acetate, formate, methanol, and acetone.

**Conclusion:**

No discrete signals suggesting a difference between remedies and controls were seen, via high sensitivity ^1^H-NMR spectroscopy. The results failed to support a hypothesis that remedies made in water contain long-lived non-dynamic alterations of the H-bonding pattern of the solvent.

## Background

The mechanism of action of homeopathic remedies has baffled practitioners and scientists for two centuries. A widely accepted premise of those doing research in this field is that if remedies are more than placebos, then the process of making remedies by alternating dilution and succussion must alter the solvent, encoding in it a "memory" or "information" that biological systems can detect. Experiments attempting to measure or document solvent alteration through direct study of the physical and chemical properties of remedies have so far failed to yield any independently replicated positive effects.

Proton nuclear magnetic resonance (^1^H-NMR, or simply NMR) is among the techniques that have been used to look for differences between remedies and control samples. The term "NMR" encompasses both solvent mobility studies (results are given as a pair of relaxation times denoted T_1 _and T_2_) and analytical studies. In analytical studies, also called spectroscopy, the results are displayed as a graph or spectrum plotting concentration against a variable called chemical shift. There are also more complex applications of NMR such as imaging and two-dimensional NMR that are not relevant to the study of discrete remedy samples. The chemical shift of a proton in a molecule in a sample reflects the (time-averaged) amount of magnetic shielding provided by the electrons making up the covalent or hydrogen bond(s) in which the proton participates, with greater electron density generally correlating with lower chemical shift numbers. Chemical shifts are measured in units of parts per million (ppm) deviation from a reference shift. Recent literature reviews by Baumgärtner [1] and by Becker-Witt *et al*.[2] identified 18 published articles on the use of NMR to study remedies, of which 9 projects used NMR spectroscopy [3-11]. Of these nine, eight [3-10] reported finding differences between remedies and controls when focusing on the relative height, chemical shift, or width of one or more of the peaks due to H's in the solvent. However Aabel and coworkers' recent study [11] found no differences between remedies and controls. It should be noted that all studies had methodological weaknesses according to the criteria developed by Becker-Witt *et al*.[2]

One hypothesis to explain how UD remedies might be different from controls states that remedies contain long-lived stable clusters of solvent molecules that are not present in controls [12-14]. If this hypothesis is correct, then the H's making up H-bonds in the stable clusters would experience a bonding environment different from that of the ambient solvent, and they should generate a separate signal on the NMR spectrum. There would be one discrete signal or peak for each symmetry-distinct H in the cluster, along with the expected large peak at 4.8 ppm for the ambient water. "Unexplained" discrete peaks have never been reported in NMR spectroscopy studies of remedies, but we wondered if this might be because cluster concentrations are very small and the methodology has been insufficiently sensitive to detect them.

A technical but relevant point must be introduced here. One version of the "cluster theory" posits that once a solvent molecule becomes part of a stable cluster it will stay in that cluster indefinitely. We call such a cluster "non-dynamic". A trickier but more believable hypothesis is that solvent molecules cycle back and forth between the ambient solvent and the clusters. If only one molecule at a time leaves a cluster and it is replaced quickly by another solvent molecule, then the pattern or geometry of the cluster could be sustained without indefinitely tying up any individual solvent molecules. Exchanges of surface hydrogens could also occur rapidly without losing the cluster geometry. A cluster whose components exchange with the ambient solvent would be called "dynamic". The key concept is a parameter called "dwell time", which is the average time a molecule spends in a cluster. If the dwell time is shorter than 10^-3 ^sec or so, NMR will not be able to "see" it because the chemical shift will be the average over some milliseconds, and the discrete signals will blend back in with the ambient water signal. Dwell times are considerably shorter than 10^-3 ^sec for a variety of processes, such as ion solvation [15-17] and protein association [18-22]. Therefore NMR spectroscopy is a good method for testing the cluster hypothesis only if, as part of the hypothesis, we postulate that clusters are non-dynamic or that dwell times are on the order of several milliseconds or longer.

The project described in this article used a high-sensitivity NMR method to test this possibility, i.e., do remedies made in water contain low concentrations of long-lived non-dynamic regions of structured H-bonding? A good starting point was to quantify the detection limits of previous studies and of the present study. Without a detection limit a negative finding is hard to interpret. The methodology of Aabel *et al*.[11], which did not include individual shimming of tubes and collected the standard 16 scans per tube, might be expected to have a detection cutoff around 1 mM on a 500 MHz magnet. That is, peaks representing concentrations of H smaller than 0.001 mol/L would be lost in the noise.

This project undertook to improve the detection limit by conducting individual shimming, increasing the number of scans collected, and most importantly, utilizing a high sensitivity method called presaturation. With presaturation, the H's contributing to the 4.8 ppm peak are excited in advance in a manner which causes their contributions to nearly cancel each other out rather than combine into a huge peak. As a result the receiver gain can be increased and signals that would otherwise be overwhelmed become detectable. By combining these techniques, peaks belonging to H-containing compounds at concentrations as low as 5 μM were consistently detected.

Assuming unsplit peaks, this means that if a remedy contained a population of a particular stable cluster species at a concentration of 5 μM, the method would be able to detect it. Assuming peaks are split as doublets, the cutoff rises to 10 μM. Although the H's on an H_2_O molecule are normally NMR-equivalent, they need not be equivalent if the H_2_O is embedded in a fixed stable cluster. Then each H of an H_2_O could split the other's peak, yielding doublets. Protons on distinct H_2_O units are far enough apart that their coupling constants can be expected to be too small to generate further peak splitting. Based on this reasoning we assume doublets as the norm for a hypothetical stable cluster, and we take 10 μM as the detection cutoff. For perspective, at 10 μM, we could detect structuring if just 1 in every 10^7 ^protons were involved in a fixed H-bond.

## Methods

### Chemicals

Chemicals including 100.0% D_2_O, 100.0%-d DMSO-d6, and 98% 1,4-cyclohexanedione (C_6_H_8_O_2_) were obtained from Sigma-Aldrich [23]. Distilled deionized water was either obtained from Sigma-Aldrich or prepared on site via a purifier capable of producing 18 MΩ·cm water. Ultra-pure water was kept in tightly closed polypropylene bottles. Measurements at the point of use found that the water had conductivity no greater than 2 μS (or 0.5 MΩ·cm). The rise in conductivity occurs within minutes as ultra-pure water picks up CO_2 _from exposure to air and ions from exposure to glass, and we considered it to be unavoidable.

### Remedies

Two animal (*Sepia *and *Lachesis*), two plant (*Ignatia *and *Lycopodium*) and two mineral remedies (*Natrum Muriaticum *and *Argentum Nitricum*), all of which occur commonly in clinical homeopathy, were used for the project. Commercial remedies were obtained from Helios Pharmacy [24] and from Washington Homeopathic [25]. Both pharmacies are widely considered to make quality products that give good clinical results. Helios remedies are regarded by many to be among the best homeopathic remedies in the world. Each pharmacy provided all six remedies at the 12 C, 30 C, 200 C, 1 M, and 10 M potencies. Staff at both pharmacies made the 12 C and 30 C remedies via the Hahnemann process in washed and rinsed vials using distilled deionized water at each dilution, starting from a standard mother tincture (MT). For the 200 C potencies, a 195 C potency in ethanol-water "off the shelf" was used as the starting point for five serial dilutions and succussions in deionized distilled water. Likewise, 1 M and 10 M potencies were derived from "library" 995 C and 9995 C potencies made in ethanol-water. It was a consensus among homeopathic practitioners that despite the switch from ethanol-water to water, this was a valid way of generating 200 C and higher potency remedies. Upon receipt, commercial remedies were stored in the bottles in which they were sent, at room temperature, in a box in a dark cupboard. Each pharmacy also provided an unsuccussed water control.

We also made our own mineral remedies up to the 12 C potency starting from MT's consisting of a hand-made 1 M NaCl solution and a stock bottle of 0.1 N AgNO_3_. To make a remedy series, twelve 12 mL capped borosilicate glass vials were labeled 1 C through 12 C, and to each was added 4 mL water. Two drops of MT were added to the first and it was succussed, then 2 drops of the 1 C were added to the next vial and it was succussed, and so on. Transfers were via sterilized Pasteur pipettes. Vials and pipettes were not re-used. Succussion consisted of 120 strokes of forcefully pounding the closed vials held in the fist against a rubber mouse pad on a counter top. Succussed water to be used as a control was made the same way. Over the course of the project, seven series of *Nat Mur *potencies and six of *Arg Nitr *potencies were made. Remedies were made less than 24 hours in advance of their scheduled testing times. They were placed into NMR tubes and readied for analysis within an hour of being produced. The tubes generally waited overnight in a light-resistant foil-wrapped container at room temperature before undergoing analysis.

### NMR Methodology

To prepare a sample for analysis, a borosilicate glass NMR tube rated for 500 MHz (Wilmad Lab Glass [26]) was primed with 50 to 90 μL of locking agent (either D_2_O or DMSO-d6) and 20 μL of a dilute water solution containing a known concentration of a marker molecule (markers tried were acetone (CH_3_COCH_3_) and 1,4-cyclohexanedione (C_6_H_8_O_2_)). The remedy or control sample was then added to fill the tube to the 700 μL mark, followed by gentle tilting and turning to mix. The marker served several purposes: it provided a reference line for zeroing the chemical shift scale, it provided a reference peak for comparing concentrations, and its sharpness and shape gave feedback about the accuracy of the shimming process. By carefully varying the marker concentration we also determined the method's limits of detection. [C_6_H_8_O_2 _was considered an "ideal" marker in that it met all of these criteria: it is available cheaply at high purity; it dissolves in water without altering pH and does not evaporate over time; its ^1^H-NMR spectrum has a single unsplit line; its unique peak occurs at a distinctive location far from the water peak and is not easily confused with other common peaks (2.77 ppm); and it will not normally occur as a contaminant or from other sources, so one can be sure its concentration is exactly what one intends.]

Proton NMR spectra were obtained at the Department of Chemistry Instrumentation Facility at M.I.T. ("DCIF"). All spectra reported here were obtained on a Varian INOVA 500 tuned to 499.759 MHz and equipped with an inverse probe. Tubes were run with the temperature clamped at either 20°C or 21°C and were not spun. Shimming was done manually for Z1 through Z7 and for first and second order XY magnets, using the lock signal. Presaturation used the standard presat pulse sequence (satdly = 1.5 sec), with the optimal presat frequency being determined to within 0.1 Hz via an array method. Locking, shimming and presat frequency optimization were repeated each time a tube was inserted. Between 128 and 200 transients were collected for each sample. Start-to-finish time for each tube was typically around 35 minutes. Spectrum analyses were done via the standard Varian programs running on an SGI workstation provided by DCIF.

### Randomization

Randomization and blinding are among the recommendations in Ref. [2] for how to conduct high quality studies on homeopathy. We did not conduct strict randomization, but we did intentionally "mix up" the samples in some respects. We labeled all NMR tubes at the outset and we deliberately changed around which tubes were used for controls and for remedies, in case tube-specific effects were to occur. On most days when we were analyzing remedies we included at least one control, and we always ran the control neither first nor last, in case a time-related trend or drift were to influence the results. Our plan was this: if any signals appeared which seemed to be occurring significantly more often in remedies than in controls, the studies would be followed up with strictly randomized, blinded trials to verify those particular signals. If no significant differences were found, then strict randomization could not alter the outcome, and the follow-up step would be unnecessary.

## Results

We analyzed a total of 57 remedy samples, 5 succussed water controls, and 41 unaltered water controls. Of this total, 28 samples were non-commercial (i.e. made on site) *Nat Mur *and *Arg Nitr *remedies of potency 6 C to 12 C. Because the details of the protocol evolved over the course of the project, it could be argued that this total represents the combination of many different experiments. Realizing this, we made a point toward the end of the project, of applying what we considered to be our best methodology to screen a set of what were arguably the best remedies. Specifically, we screened 18 Helios remedies, namely the 12 C, 30 C and 10 M potencies of the six MT's. The screening protocol consistently used 70 μL of D_2_O for locking and concentrations of 12.5 μM or less of C_6_H_8_O_2 _as marker. These 18 trials are included in the 57 for the purposes of discussion. Spectra can be conveniently split into those obtained during 2002 (listed by sample and date in Table 1, Additional File 1) and the Helios screening run (listed in Table 2, Additional File 1).

### Sensitivity

As indicated above, when marker concentration was 5 μM of H or greater (e.g. 0.62 μM of C_6_H_8_O_2_), a peak was always seen above noise, using the 3σ criterion [27]. This included four samples where the concentration was just 5 μM. A peak was seen in two of three samples where the concentration was 4 μM and in neither of two samples where it was 3 μM. Thus 5 μM was taken as the detection cutoff for this methodology.

### Expected and unexpected signals

Expected signals that were seen in all spectra included the large water signal peaking at 4.81 ppm, which dominated the spectrum despite presaturation. The water signal is so dominating that small peaks between approximately 4.4 and 5.2 ppm could be "lost" in it, and this interval must be viewed as an inaccessible region of the spectrum for our methodology. Marker peaks for CH_3_COCH_3 _and C_6_H_8_O_2 _were observed respectively at 2.22 [28] and 2.77 ppm. ^13^C satellites were seen as expected when marker concentrations were high enough and are not listed separately in Table 3, Additional File 1. When DMSO-d6 was the locking agent, a residual DMSO-d5 quintuplet centered at 2.68 ppm was always present.

The focus of this project was to look for discrete peaks other than these expected peaks. Combining the 103 spectra, there were 35 positions where a discrete signal occurred that was not among these expected signals. These positions are listed in Table 3, Additional File 1. Signals that had the same structure (i.e. singlet, doublet, etc.) and occurred at the same position (within ± 0.01 ppm) in multiple spectra were assumed to have the same genesis. (This assumption could be challenged, but it would not affect our overall conclusions.) We therefore examined each of the 35 "unexpected" positions to see if we could offer an explanation for why signals occurred there.

### Artifacts

Fifteen of the 35 signals were classified as artifacts, i.e. machine-generated spectrum "glitches" that were unrelated to the sample. A signal was dismissed as artifact if it could not be phased consistent with the marker peak. Artifacts were further classified as either "consistent" or "intermittent". The consistent artifacts occurred in more than half of all samples and throughout the eighteen months when our data was collected. Consistent artifacts were seen at 3.60, 8.17, and 11.53 ppm. Signals at positions 8.17 and 11.53 were explained as mid-spectrum artifact and as a "reflection" of the water signal respectively (Δ = 8.17 - 4.81 = 3.36, reflection across midpoint is at 8.17 + Δ = 11.53), but no rationale for the 3.60-ppm artifact was identified. We labeled the consistent artifacts as C1, C2, and C3.

Intermittent artifacts were labeled R1 through R12 and are listed in Table 3, Additional File 1. These artifacts tended to be small, often little more than bumps or wiggles in the baseline. With the marker peak phased to go up (i.e. wholly above the baseline), artifacts might be entirely below the baseline (downgoing), entirely above (upgoing), or both (biphasic). An artifact that occurred repeatedly could have different behaviors in different spectra. Table 3, Additional File 1 indicates whether each artifact was downgoing (d), biphasic (b), or upgoing (u), or whether more than one behavior was seen. The criterion for classification as artifact was that a signal was downgoing or biphasic in at least some of the spectra where it was seen.

Half of the intermittent artifacts were seen in only one spectrum each. Those that occurred repeatedly demonstrated a "burst" pattern in the sense that they were present for spectra collected on a single day or during an interval of weeks or months, but were not seen at other times. For instance, the artifact R5 occurred only on 4-Jun-03 and was seen in every spectrum obtained that day (the control as well as the remedies: Table 2, Additional File 1).

### Contaminants

Signals that were consistently upgoing could be artifacts or they could be measuring something actually present in the sample. Among such signals, some were evidence of contamination by small organic molecules. Seven signals were positively identified, and an eighth was given a probable assignment.

Zacharias [29,30] raised the issue that some amount of contamination was probably unavoidable in remedies, and that it could affect the outcome of studies of remedies. In our spectra signals representing contamination by small organic molecules were frequently seen. Non-commercial remedies and controls often contained acetate (CH_3_COO^-^, 1.90 ppm [28,31]) or formate (HCOO^-^, 8.44 ppm [28]) ions at concentrations ranging from barely detectable (1.7 μM for acetate, 5 μM for formate) to 30 μM. Long soaking of the NMR tubes in water between uses and exercising extra care to avoid hand contact with the remedies decreased but did not eliminate the occurrence of these two contaminants. Lactate (CH_3_CHOHCOO^-^, 1.32 ppm [31]) occurred at barely detectable levels in 3 non-commercial samples. The fact that its signal is a doublet with the characteristic 5 Hz coupling constant assists with the identification, and improves one's confidence that one is seeing a real signal and not just noise that happens to occur at the chemical shift of lactate. The α hydrogen signal of lactate, expected around 4.2 ppm, is a quartet and would be predicted to have 1/4 the height of the methyl peak. The quartet would have been a nice confirmation of the lactate identification, but it could not be seen above noise.

One signal, an upgoing singlet at 1.28 ppm, occurred in all but one spectrum run using DMSO-d6 between April and November 2002, and did not occur in spectra run using D_2_O or outside this time interval. We pegged it as coming from a contaminant in the DMSO-d6, which was not present in different bottles of DMSO-d6 that were used before April and after November. Its base was broader by a factor of four than the other singlets seen, and despite its trace size we could often make out that it had a symmetric stepped shape like a ziggurat. A good guess, which fits all of these facts, is that it comes from ethylmethylsulfoxide-d7 (CD_3_SOCD_2_CD_2_H), which could plausibly be introduced during the manufacture of DMSO-d6 (CD_3_SOCD_3_). We did not find anywhere listed the chemical shift of ethylmethylsulfoxide, but the ethyl's methyl group of the very similar molecule ethylmethylketone resonates at 1.26 ppm [28] in D_2_O. We gave it the identifier 'X' in Table 3. A DMSO-d6 control run on 1-Aug-02 yielded two peaks in the vicinity of 1.3 ppm, one of which may have been 'X'.

For the 18 Helios remedies screened with the C_6_H_8_O_2 _protocol, results are given in Table 2, Additional File 1. All but one had measurable quantities of ethanol, with concentrations typically around 300 μM but in one case as high as 3.6 mM (range 116 – 3632 μM, median 310 μM). The ethanol signal, a triplet at 1.17 ppm paired with a quartet at 3.65 ppm, was unmistakable. The ratio between the peak areas of ethanol's methyl triplet at 1.17 ppm and its methylene quartet at 3.65 ppm is theoretically 3:2. In Table 2, Additional File  1the range of ratios is 1.37 to 1.66. The proximity of the variably-shaped artifact near 3.60 ppm sometimes interfered with accurate determination of the area of the methylene signal, and ethanol concentrations were taken to be 1/3 of the 1.17-ppm peak area. Repeat measurements of a single sample showed that the concentration figures obtained this way have an experimental error of 5 – 10 %.

The 18 Helios remedies of Table 2, Additional File 1 all contained CH_3_COO^- ^(range 22 – 214 μM, median 55 μM) and HCOO^- ^(range 8 – 75 μM, median 44 μM). None contained detectable lactate, but we sometimes saw acetone or methanol (CH_3_OH, 3.34 ppm [28]). Six of the 18 contained detectable acetone (range 3 – 21 μM, median 5 μM) and a different but overlapping set of six held detectable methanol (range 2 – 10 μM, median 4 μM). Because the samples prepared on site never contained detectable ethanol, methanol, or acetone (excepting the added acetone markers), and all samples were analyzed by the same procedure, it is safe to conclude that the Helios samples came with these contaminants. While we admit that our procedures and lab technique were apparently introducing some extraneous acetate and formate, the Helios remedies' levels of these ions typically ran higher than the levels seen in remedies prepared on site (medians of 55 and 44 versus a maximum of about 30 for on site remedies). We deduce that the source of these contaminants in Helios remedies was at least partially from the remedies themselves, i.e. most or all of the Helios remedies came with some acetate and formate in them. The three Helios remedies that we examined using DMSO-d6 contained ethanol, acetate, and formate as well, and one had methanol, but for those runs we did not add a measured marker and precise concentrations were not determined (Table 1, Additional File 1). Of the eight Washington Homeopathic remedies that we analyzed and the Washington control, all had ethanol and acetate, all but the control had formate, seven had acetone, two had methanol, and six had lactate. The high rate of lactate occurrence in Washington's samples (6 of 9 vs 3 of 94 for non-Washington) was not due to chance (p < 10^-6^), but the fact that the six runs exhibiting lactate were all done on one day with no non-Washington control means that we cannot rule out an extraneous source for the lactate.

### Unexplained signals

Twelve positions for upgoing singlets could neither be ruled out as artifacts nor assigned definitely to any known small organic contaminant. These are the "unexplained" peaks, labeled U1 through U12 in Table 3, Additional File 1. Six of the unexplained group occurred in just one spectrum each. Those that occurred repeatedly exhibited the same kind of temporal burst distribution that was seen for the intermittent artifacts. (The only exception was U1, which was seen on 2-Jan-02 and again on 20-May-02; this may be an example where two different signals coincidentally had similar shapes and chemical shifts and were lumped together.) The ppm range was from 0.47 to 10.60; among those that occurred more than once the range was 0.47 to 6.80. The three upfield-most (i.e. lowest ppm) signals were "broad", i.e. width at half height was between 0.02 and 0.1 ppm, in contrast to the typical "sharp" singlet whose width at half height was 0.002 to 0.004 ppm.

For each of the unexplained signals and artifacts we made a 3 × 2 matrix of their occurrence vs non-occurrence in remedies, succussed controls, and unsuccussed controls. None of the matrices had a p-value below .05. The occurrence counts and p-values are listed in Table 3, Additional File 1.

### Spectrum example

**Figure 1:**
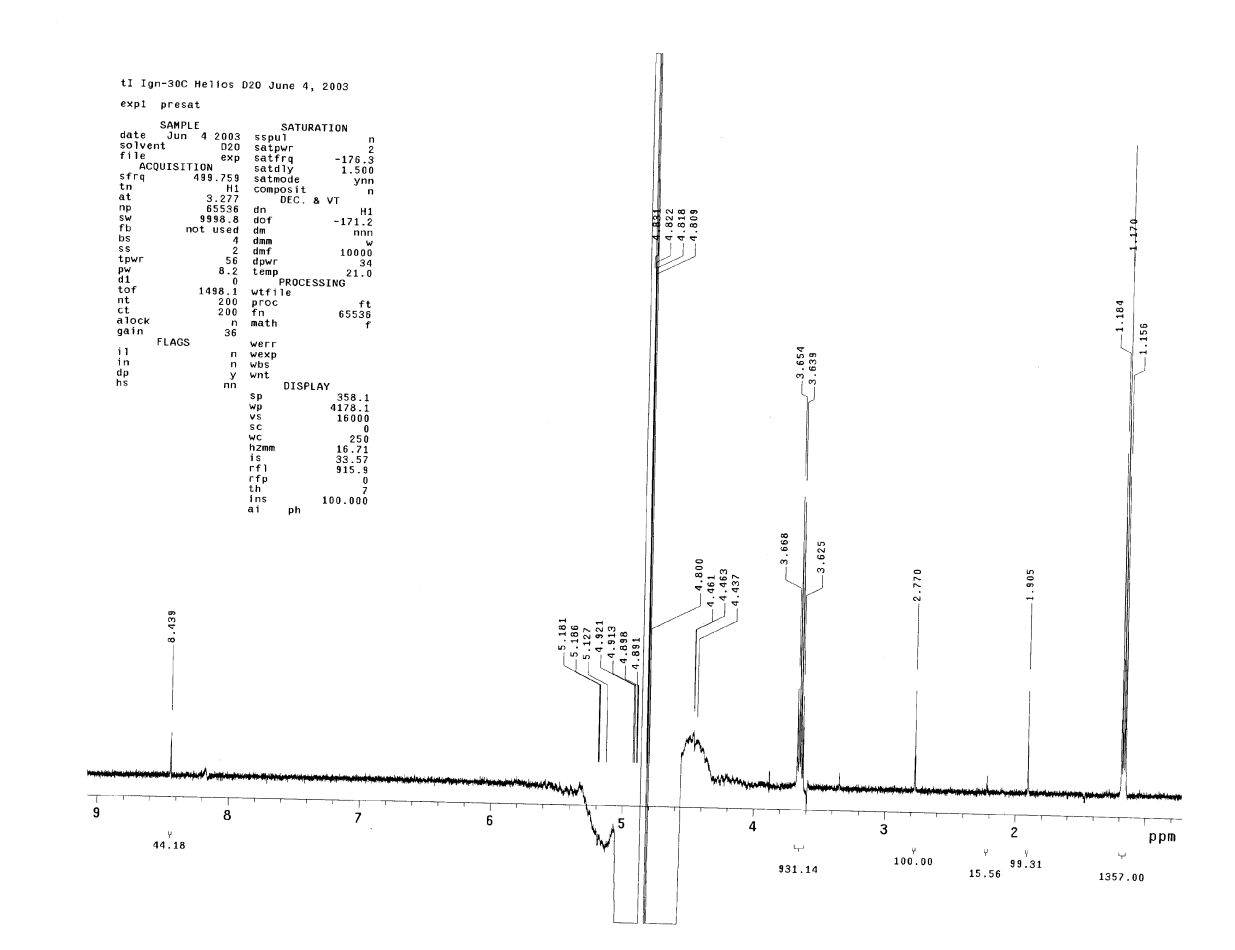
^1^H-NMR Spectrum of *Ignatia*-30 C (Helios): expansion of 1 – 9 ppm region. Key to peaks [position – interpretation]: 1.17 – ethanol, methyl triplet; 1.47 – artifact (R4); 1.90 – acetate; 2.22 – acetone; 2.77 – C_6_H_8_O_2 _(1,4-cyclohexanedione marker); 3.35 – methanol; 3.60 – artifact (C1); 3.65 – ethanol, methylene quartet; 3.88 – artifact (R5); 4.3 to 5.3 – water (presaturated); 8.17 – artifact (C2); 8.44 – formate; 11.53 (not shown) – artifact (C3).

**Figure 2:**
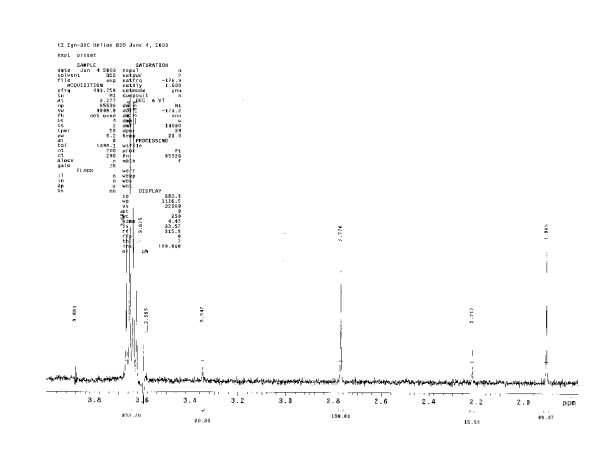
^1^H-NMR Spectrum of *Ignatia*-30 C (Helios): expansion of 1.8 – 4.0 ppm region. Key to peaks [position – interpretation]: 1.90 – acetate; 2.22 – acetone; 2.77 – C_6_H_8_O_2 _(1,4-cyclohexanedione marker); 3.35 – methanol; 3.60 – artifact (C1); 3.65 – ethanol, methylene quartet; 3.88 – artifact (R5).

Figure 1 shows a portion of the spectrum of Helios' *Ignatia*-30 C sample (Line 27 of Table 2). Figure 2 shows a magnification of the same spectrum between 1.8 and 4.0 ppm. Numbers below the x-axis represent integrated peak areas, relative to C_6_H_8_O_2 _peak at 2.77 ppm being set to its known value of 100 μM of H. The complex signal between approximately 4.3 and 5.3 ppm is the presaturated water signal. This spectrum contains five contaminants, the three consistent artifacts (artifact at 11.53 not shown), and two intermittent artifacts. Note that the artifacts are biphasic, i.e. have a component below as well as above the baseline, whereas all of the signals due to actual molecules have signals that stay above the baseline.

## Discussion

### Contaminants

Concerning the organic contaminants, acetate, formate and lactate are present on human skin and can be introduced at the trace levels seen here through ordinary handling. Acetate and formate derive respectively from the β- and α-oxidation of long-chain fatty acids by fibroblasts [32], and lactic acid is the principal organic component of eccrine sweat (and is found at higher concentrations on the palms [33]). They are frequently encountered in high sensitivity work. Methanol and acetone are commonly used for rinsing glassware, and a reasonable but unprovable guess is that they represent a residuum from a vial rinse. A plausible but again unprovable guess is that the source of the ethanol was incomplete flushing after prior use of the vials to contain ethanol-based remedies, or perhaps ethanol was also used in the rinsing process. The source of the ethanol for the 200 C and higher potencies is not the 195 C or other library potency: if there were no other source of ethanol, five 1:99 dilutions in water would reduce the level to at most 17 × 10^-10 ^M.

Despite the implication that commercial remedies may be "impure", we consider the levels of detected impurities to be extremely low. Large doses of methanol are hepatotoxic, but the trace amounts here represent no threat to health. Our findings certainly support the widely accepted view that homeopathic remedies are safe. We also believe that the contaminants found should be unlikely to interfere with remedies' clinical effectiveness. The median value of 310 μM corresponds to less than one part ethanol in 50,000 parts water. Working in the early 19^th ^century, Hahnemann would have used locally obtained well water or spring water to make remedies, and his water would have been far less pure than our most contaminated sample. (Hahnemann also used wine and brandy to make his remedies – hardly the precision solvents of the modern lab.) While high purity solvents are necessary for scientific validity and reproducibility, there is no *a priori *reason to think they matter for remedies' effectiveness. Indeed, one of the hypotheses on the mechanism of homeopathy is that the "information" is in the geometry of the solvation shells of the low levels of impurities that are present in the solvent, or in some structuring of how solvent impurities group together or interact with each other.

### Unexplained peaks

Six of the unexplained signals occurred in just one spectrum each and could easily be artifacts that happened to be in phase with the rest of the spectrum. There is little to be gained by trying to explain these one-time events, five of which were from unsuccussed controls. The signals U1 to U3, being broad and upfield, could be consistent with complex aliphatic mixtures such as finger grease on the outside of the NMR tubes [28]. We have no conjecture as to the identity of U5 (2.80 ppm, 3 occurrences) or U6 (4.12 ppm, 2 occurrences).

Signal U8 at 6.80 ppm occurred in 7 of 8 D_2_O runs starting in November 2002, which was when a fresh bottle of D_2_O was brought into use, and it was not seen in the DMSO-d6 runs from the same period. This pattern suggests it was a contaminant in the D_2_O, even though it disappeared when the same D_2_O bottle was used in April 2003. There was also one sporadic occurrence in April 2002 (a DMSO-d6 spectrum). A likely assignment is quinone (p-benzoquinone, C_6_H_4_O_2_), whose spectrum consists of one singlet at 6.80 ppm [34]. Quinone sublimes at room temperature, and the approximately one micromole of quinone in the 20-mL D_2_O bottle may have simply evaporated during four months.

In Table 3, Additional File 1 we also list the occurrence counts for "any unexplained signal" in remedies and in controls, and its p-value of 0.08. Lest this appear as a "trend", we point out that the reason for this relatively low p-value is that the "trend" was for controls to show more unexplained signals than remedies. We do not believe the difference is meaningful.

### Other aspects of ^1^H-NMR spectra

We found no discrete signals that occurred significantly more frequently in remedies than in controls. Does this mean that NMR spectroscopy cannot show differences between remedies made in water and controls? Earlier NMR studies focused not on additional signals but on peak positions and shape. For samples in water there is only one peak, the water peak at 4.8 ppm, along with the expected peaks for marker and/or DMSO-d5. We did not examine the shape of the water signal. Presaturation completely destroys any information that may have been present in the water signal shape, and the shape of the water signal after presaturation is sensitive to so many variables that it would be hard to track down the effect, if any, of solvent alteration. Still, this is an avenue that could conceivably be pursued. Similarly, we indicated that any information in the range of 4.4 to 5.2 ppm was essentially lost. It might be possible to examine this range closely, perhaps by subtracting off a smoothed spectrum, to look for signals between 4.4 and 5.2. We have not attempted this. We were primarily looking for separate peaks that could be from long-lived stable clusters, and did not find any. For this reason we couch our results carefully by saying merely that "no discrete signals suggesting a difference were seen."

### Implications for homeopathic mechanism

The absence of any evidence of stable H-bonds above the 5 μM or 10 μM detection limit, in 57 remedy samples, certainly casts doubt on the hypothesis that remedies in water might contain regions of long-lived non-dynamic H-bond structuring. Is this hypothesis salvageable? We do not believe so. We made a point of screening low, medium and high potency remedies, as well as remedies from six common MT's. While it could be argued that even 10% DMSO could disrupt H-bonding and thereby denature (i.e. destroy) H-bond structure [35], this cannot be said about D_2_O. Nor do we believe that the addition of the marker could have interfered with the detection of structuring. Consider that Helios's remedies, reputedly among the best anywhere, contained comparable or higher concentrations of organic contaminants to start with, than we added. If one wants to maintain the position that homeopathy works (i.e. clinical effects are attributable to it), and that Helios's and Washington's remedies work, then the fact that their remedies come with traces of small organic molecules tells us that such traces cannot be something that interferes with homeopathy's mechanism. Thus it should not "hurt" a remedy to add a tiny bit of organic marker.

Could there be signals from fixed H-bonds but we didn't see them because they fell in the inaccessible 4.4- to 5.2-ppm region? The chemical shift of an H in a fixed O-H - - O setup depends on the O - - O separation and the details of the geometry, but studies of ice [36], organic H-bonds [37,38] and *ab initio *simulations [39,40] all show that chemical shifts in the range 7 – 16 ppm can be anticipated. For this reason we do not believe that obliteration by the water signal of the 4.4- to 5.2-ppm interval represents important data loss. Our 103 spectra were particularly sparse in signals in the downfield (i.e. > 7 ppm) region. Besides the two "explained" artifacts C2 and C3 and the identified contaminant formate, they contained just 3 single-occurrence downfield unexplained signals and just 3 occurrences of downfield intermittent artifacts. Thus our results were very different from the constellation of downfield signals predicted by the non-dynamic stable cluster hypothesis.

We have ruled out, or more precisely we have rendered highly improbable, only this one hypothesis on the nature of the "active ingredient" of homeopathy. Although a positive finding would certainly have been interesting, our negative findings should not be taken as evidence against clinical homeopathy. In particular, the possibility of dynamic alterations of solvent structuring remains open, but this hypothesis will need to be studied by methods that take a much faster "snapshot" of what is going on in samples. Finally, there are also several non-cluster-based hypotheses that have been proposed to explain homeopathy. These include isotopic patterning, coherence, and chaos-based explanations [41]. These explanations do not require any long-lived H-bonds and do not predict that "unexpected" discrete peaks would be seen in the NMR spectra of remedies.

## Conclusion

We used a high sensitivity ^1^H-NMR spectroscopy method to look for discrete signals that could provide evidence of pockets of fixed H-bonding in water-based homeopathic remedies. No such evidence was found. The method did reveal the presence of some small common organic molecules, at levels deemed too low to be problematic. We hope that we have made a contribution to homeopathic research, both by answering a particular question, and by setting a standard for quality hypothesis-driven research that others will be inspired to follow.

## Competing interests

The author(s) declare that they have no competing interests.

## Pre-publication history

The pre-publication history for this paper can be accessed here:



## Supplementary Material

Additional file 1 Table 1: Results of runs during 2002, Table 2: Results of Helios remedies screening, Table 3: List of peak positions Click here for file
